# Prognostic value of histopathologic features with respect to disease course in children with juvenile dermatomyositis

**DOI:** 10.1186/1546-0096-10-S1-A61

**Published:** 2012-07-13

**Authors:** Annette Y Lopez-Martinez, Lili Miles, Bin Huang, Kevin Bove, Daniel J Lovell

**Affiliations:** 1 Cincinnati Children's Hospital Medical Center, Cincinnati, OH, USA

## Purpose

Evaluate pre-treatment muscle biopsies of patient’s with probable or definite Juvenile Dermatomyositis (JDM) using the Wedderburn score to predict clinical outcomes.

## Background

JDM is the most common idiopathic inflammatory myopathy in children. The Wedderburn scoring system is used to assess JDM muscle biopsies and categorizes muscle involvement into 4 domains: inflammatory, vascular, muscle fiber and connective tissue fibrosis (maximum scores: 12, 3, 10, and 2, respectively) and a 10-cm VAS measuring overall abnormality. A 10 cm VAS for chronicity (endomysial fibrosis, capillary loss and central nuclei) was also measured.

## Methods

In this retrospective cohort study JDM patients diagnosed from 1995-2008 underwent pre-treatment muscle biopsy guided by soft tissue MRI at CCHMC. Children had probable or definite JDM (Bohan and Peter criteria) and disease duration ≥ 2 years. Disease was classified as “limited” or “chronic” based on treatment duration and chronic disease was subdivided into “nonulcerative” or “ulcerative” (cutaneous or gastrointestinal ulcers) (Crowe et al, 1982). Demographics, disease manifestations, complications and treatment duration were collected. Severity of muscle weakness was assessed at presentation, 2 years, and last visit. Muscle biopsies were evaluated jointly by 2 pathologists using the Wedderburn scoring system. Wilcoxon Sign Rank Test and Spearman Correlation Coefficient (CC) were used.

## Results

22 patients were studied with mean (median) age at diagnosis 7.4 ys (6.5), follow up duration 49.5 months (47.5) and treatment duration 44.1 months (35). 2 patients had limited disease and 20 chronic disease, all were nonulcerative. Patients had mild or moderate weakness at first visit and no or mild weakness at 2 ys and final visit. Mean (median) Wedderburn scores were as follows: inflammatory 6 (6.5), vascular 0.9 (1), muscle fiber 5.8 (6), connective tissue fibrosis 1 (1), VAS overall 3.6 (3.2), and VAS chronicity 2.1 (1.1). The connective tissue fibrosis domain and the VAS chronicity score had the strongest correlations with muscle weakness at the final visit and moderate correlations with muscle weakness at all visits. (Table 1) Statistical significance was limited by the small sample size. There were no patients with very poor outcomes (e.g. death, ulcerative disease).

**Table 1:** 

Domain or VAS	Weakness 1^st^ visit	Weakness 2 years	Weakness final	Disease type	Treatment duration
Connective tissue fibrosis	CC=0.4	CC=0.4	CC=0.4	CC=0.01	CC=0.1
	p=0.04	p=0.06	p=0.06	p=1	p=0.5

Muscle	CC=0.2	CC=0.03	CC=0.06	CC=0.3	CC=0.3
	p=0.4	p=0.9	p=0.8	p=0.2	p=0.2

Inflammatory	CC=03	CC=-0.1	CC=0.1	CC=0.2	CC=0.3
	p=0.2	p=0.7	p=0.6	p=0.4	p=0.2

Vascular	CC=0.3	CC=0.1	CC=0.3	CC=0.2	CC=0.4
	p=0.1	p=0.5	p=0.2	p=0.4	p=0.1

Overall biopsy score (VAS)	CC=0.2	CC=0,1	CC=0.2	CC=0.2	CC=0.02
	p=0.3	p=0.6	p=0.5	p=0.3	p=0.9

	CC=0.3	CC=0.3	CC=0.4	CC=0.1	CC=0.2
	p=0.1	p=0.1	p=0.07	p=0.7	p=0.3

## Conclusion

The Wedderburn connective tissue fibrosis domain score associates with severity of muscle weakness. No other significant correlations. The VAS chronicity score is moderately associated with final muscle weakness, suggesting endomysial fibrosis, capillary loss and central nuclei in nonmyopathic fibers in pre-treatment muscle biopsies as predictors of disease outcome. Table 1. Results of the Wedderburn score and correlation with weakness and clinical data.

## Disclosure

Annette Y. Lopez-Martinez: None; Lili Miles: None; Bin Huang: None; Kevin Bove: None; Daniel J. Lovell: None.

